# Trace element contamination in the mine-affected stream sediments of Oued Rarai in north-western Tunisia: a river basin scale assessment

**DOI:** 10.1007/s10653-021-00887-1

**Published:** 2021-03-26

**Authors:** Jamel Ayari, Maurizio Barbieri, Yannick Agnan, Ahmed Sellami, Ahmed Braham, Faouzi Dhaha, Abdelkarim Charef

**Affiliations:** 1 National Office of Mines, 24 rue 8601 La Charguia, 1080 Tunis Cedex, Tunisia; 2 Dip. Scienze Della Terra, Università Di Roma “La Sapienza”, P.Le Aldo Moro, 5-00185 Roma, Italy; 3 Earth and Life Institute, Université Catholique de Louvain, 1348 Louvain-la-Neuve, Belgium; 4 Université de Carthage, Sidi Bou Said, Avenue de La République, 1054 Tunis Cedex, Tunisia

**Keywords:** Trace elements, Contamination, Source, Spatial distribution, Stream sediments, Oued Rarai basin

## Abstract

High-quality and accurate environmental investigations are essential for the evaluation of contamination and subsequent decision-making processes. A combination of environmental geochemical indices, multivariate analyses and geographic information system approach was successfully used to assess contamination status and source apportionment of trace elements (Ag, As, Cd, Cr, Cu, Hg, Ni, Pb, Sb, V and Zn) in surface stream sediments from the Oued Rarai basin in north-western Tunisia, containing various metal and metalloid ores. The contamination level reported in this study indicates a non-negligible potential ecological risk, mainly related to sediment transport along the river. Antimony (concentrations ranged from 0.02 to 297 mg kg^−1^ and Igeo > 5), arsenic (from 0.5 to 1490 mg kg^−1^ and Igeo > 5), lead (from 2.9 to 5150 mg kg^−1^ and Igeo > 5) mercury (from 0.05 to 54.4 mg kg^−1^ and Igeo > 5) and silver (from 0.05 to 9.4 mg kg^−1^ and Igeo > 5) showed the most crucial contamination. Besides, potential ecological risk index values were maximum for arsenic with a median of 302, indicating a very high to serious ecological risk (> 160). Results from correlation analysis and principal component analysis revealed three main geochemical associations related to lithologic, tectonic and anthropogenic sources. V, Cr and Cu mainly originated from natural bedrock and soil. Ag and Cd were more controlled by both natural and mining enrichments. Mercury and Pb were mostly influenced by the ancient ore-related activities at the Oued Rarai site and north-east–south-west trending faults. Finally, Sb, As, Ni and Zn were largely controlled by the siliciclastic continental Neogene sequences. Finally, the physical and chemical dynamics of the watershed system, lithological properties, mineralisation, tectonic settings and mobilisation of subsurface sediments largely controlled both concentrations and spatial patterns of trace elements in the study basin. These results need to be considered in the strategies of suitable environmental management at former and current mining sites in north-western Tunisia.

**Supplementary Information:**

The online version contains supplementary material available at 10.1007/s10653-021-00887-1.

## Introduction

Historical metal mining districts have been generating significant quantities of mining wastes, which have been routinely discharged into the river systems (Clement et al., 2017; Li et al., 2019; Macklin et al., 2006; Nguyen et al., 2020; Salomons, 1995). Many studies have shown that nearly 90% of trace metal contaminants in a river system are usually associated with sediments (Argyraki et al., 2018; Hudson-Edwards et al., 2001; Hudson-Edwards et al., 2001; Meybeck & Helmer, 1989; Nguyen et al., 2020), and between 10 and 60% of the sediments discharged into the river systems can be deposited and stored within the river channels and on the floodplains bordering the channels (Goodbred & Kuehl, 1998; Owens et al., 1997).

Nowadays, contaminated sediments from river channels and floodplains continue to be a potential secondary source for trace element contamination in the hydrographic systems (Axtmann & Luoma, 1991; Clement et al., 2017). The contaminated sediments stored on floodplains can be remobilised and returned to the water–sediment interface, particularly during high-flow conditions (Lecce & Pavlowsky, 1997; Matys Grygar et al., 2014). The accumulation of trace elements in alluvial soils and sediments can have adverse effects on human livelihoods and sustainable development.

In northern Tunisia, mining of Pb–Zn ore deposits and associated activities have resulted in the discharge of important quantities of mining wastes into the fluvial systems (e.g. Jemmali et al., 2013; Sainfeld, 1952; Slim-Shimi & Tlig, 1993). Environmental studies on the impacts of these former mining activities have been conducted on the transfer and mobility of trace elements in mining wastes, soils and sediments (e.g. Souissi et al., 2013; Daldoul et al., 2015; Pascaud et al., 2015). In general, the retention capacity of soil for trace elements increases in tandem with an increasing pH, with the expected maximum being around circumneutral. Exceptions are As, Mo, Se, V and Cr, which are commonly more mobile under alkaline conditions (Sbarbati et al., 2020; Sappa et. al., 2019; Violante et al., 2010). Neutral pH and the high carbonate content play an important role in trace elements stability, in such a way that there is no transfer of soluble trace elements, the elements beings only mobilised in particulate form (Boussen et al., 2013; Pascaud et al., 2015).

Matys Grygar and Popelka (2016) reported that the settling of particles from flowing water (i.e. the distribution of solid particles between suspended load and bedload) and the variable lithology of deposits in individual fluvial environments are the primary factors driving the variability of risk–element concentrations in fluvial sediments. Finally, the biogeochemical processes can modify the sediment composition during weathering, pedogenesis and further transformations of the finest particles transported by the river in the aftermath of flooding events (Luis et al., 2019; Matys Grygar & Popelka, 2016).

Previous studies (e.g. Mlayah et al., 2009; Ayari et al., 2016) in another abandoned Pb–Zn mining site in northern Tunisia (80–100 km far from this studied site) highlight the elevated concentrations of potentially toxic trace elements released in the nearest watercourses of the past mining and metallurgical (As, Cd, Pb and Zn) as well as agricultural activities (Cu and As).

In Tunisia, the Mejerda basin is one of the most important agricultural sectors (Guellala et al., 2012). It has a strong need for irrigation water. During rainy periods, Oued Mejerda River and its tributaries are the primary sources to irrigate the vegetation (Guellala et al., 2012).

**Fig. 1:**
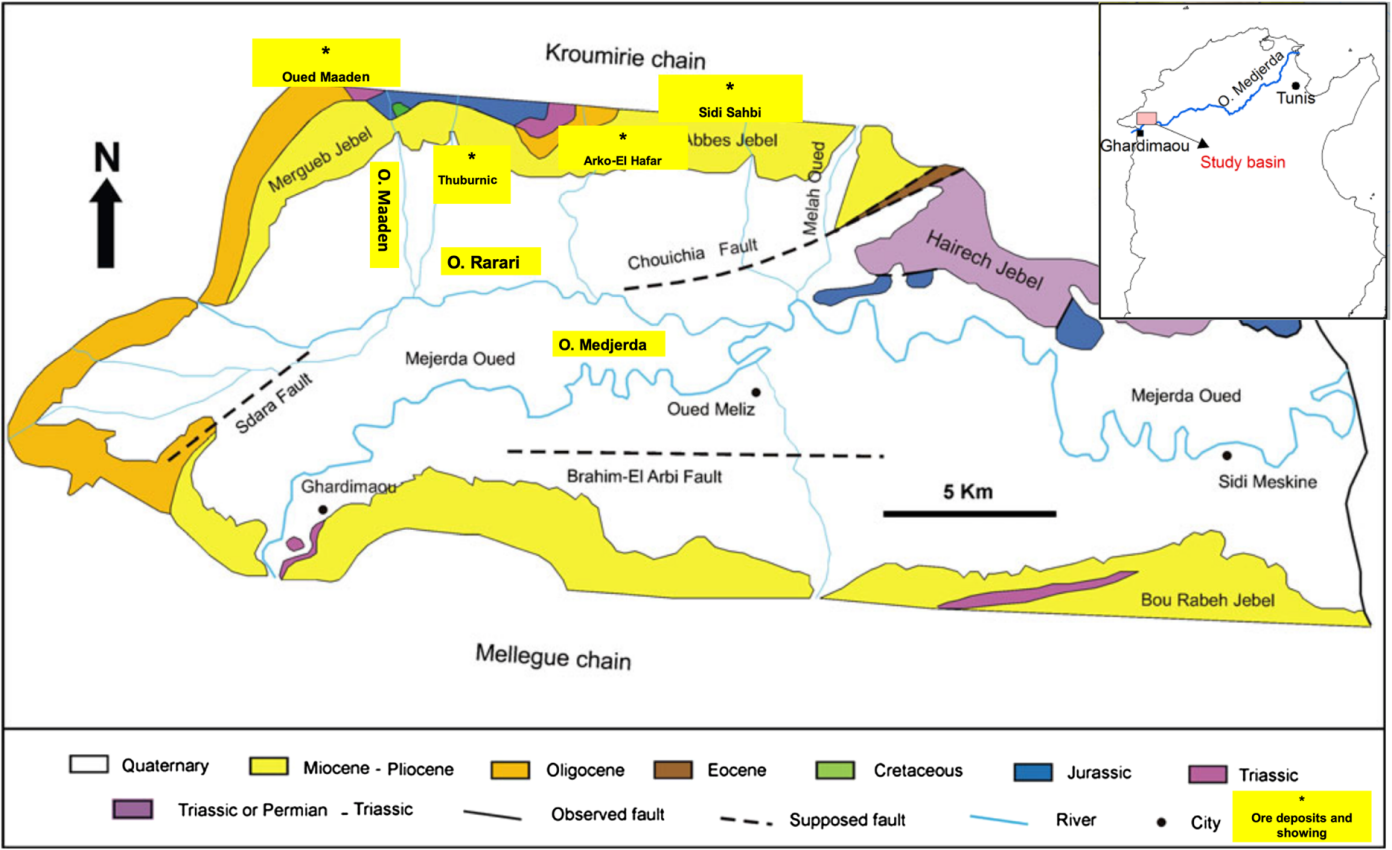
Geological and metallogenic maps of the study basin ( modified from Guellala et al., 2012)

**Fig. 2:**
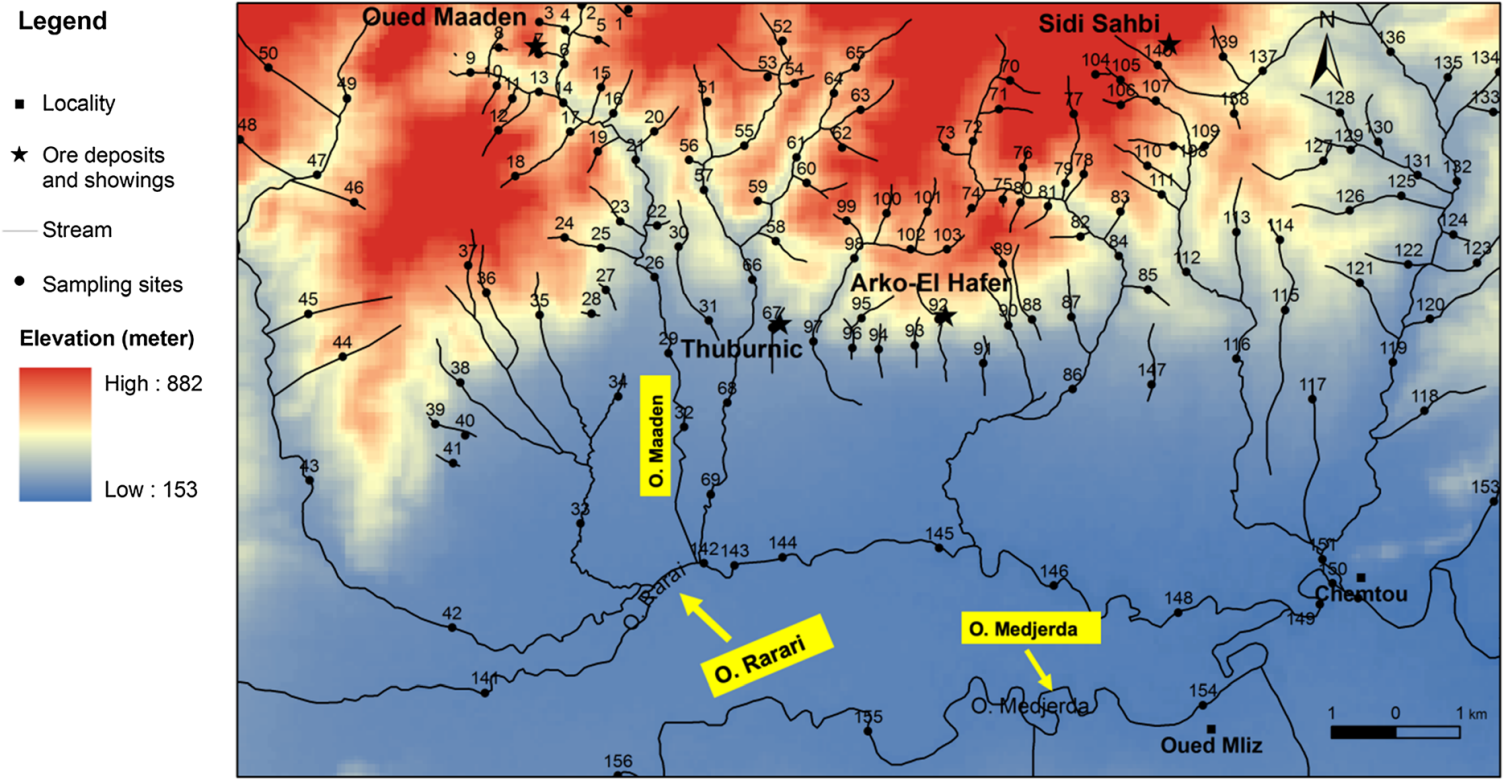
Location of the sampling sites in the study basin

No geochemical data are published on the Oued Rarai basin (Figs. 1, 2) that occupies the western area of the Medjerda river.

For that reason, the objectives of this study are:

- to assess the spatial distribution, enrichment and source apportionment of trace elements (antimony, arsenic, cadmium, chromium, copper, lead, mercury, nickel, silver, vanadium and zinc) in stream sediments from the Oued Rarai basin;
- to provide a reference for the systematic evaluation and remediation of soil contamination and sustainable management of land in the unique habitat of northern Tunisia;
- to improve the proper consideration of the transfer of trace elements under large flooding events similar to those of the Mediterranean region in recent years (Gaume et al., 2009; Llassat et al., 2010);

As guidelines, in this study, we use Dutch soil intervention values. The Intervention values for soil indicate when the functional properties of the soil for humans, plants and animals are seriously impaired or in danger of being so. Contamination of soil above the Intervention values is deemed to be severe. The soil Intervention Values apply to dry soil. The soil intervention values were first published in 2000, some of the values have been adjusted since then (Ana Payá Pérez, Natalia Rodríguez Eugenio, 2018—Status of local soil contamination in Europe, Revision of the indicator ‘Progress in the management contaminated sites in Europe’; European Commission, JRC Technical Reports, https://esdac.jrc.ec.europa.eu/public_path/shared_folder/doc_pub/EUR29124.pdf).

Dutch policy concerning contaminated land has evolved over more than three decades from a rigid assessment procedure, partly based on expert judgement, to a more flexible and tiered fitness-for-use approach with risk assessment as the underlying principle. The process to assess and appraise soil and groundwater quality follow a tiered approach, under the principle ‘simple if possible, complex when necessary’. The web-based decision support system Sanscrit has been designed to support the risk assessments within the respective policy contexts, which combine the scientific aspects of risk assessment with policy choices for protection targets and protection levels (Ana Payá Pérez, Natalia Rodríguez Eugenio, 2018—Status of local soil contamination in Europe, Revision of the indicator ‘Progress in the management contaminated sites in Europe’; European Commission, JRC Technical Reports, https://esdac.jrc.ec.europa.eu/public_path/shared_folder/doc_pub/EUR29124.pdf

## Materials and methods

### Study area

The Oued Rarai basin occupies the western area of the Medjerda River, close to the Algerian–Tunisian border, and covers nearly 467 km^2^ (Fig. 1). According to the 1992–2002 meteorological data (INM, 2003), the local climate is sub-humid, with the average annual rainfall ranging from 600 to 800 mm occurring mostly during winter and autumn as heavy storms. The average annual temperature oscillates between 14.5 °C and 18.2 °C. Similar to the Mediterranean region, the frequency of large flooding events resulting in large inundations of the floodplain has increased in recent years (Gaume et al., 2009; Llassat et al., 2010).

Morphologically, the northern part of the Oued Rarai basin comprises the north-east–south-west (NE–SW) Kroumirie chain (Tell region) and the southern part of the Ghardimaou plain, which is drained by Oued Rarai and Oued Medjerda (Fig. 2). The Kroumirie chain, part of the Nappe zone, consists of a pile of allochthonous thrust sheets formed during the Neogene tectonic event (Rouvier, 1977) with felsic plugs and mafic dikes, sills and basaltic flows of the late Miocene and Pliocene Nefza magmatic province (Jallouli et al., 2003).

The main lithologies exposed in the study basin include carbonate and siliciclastic sedimentary series ranging from the Triassic to the recent age (Fig. 1): Triassic gypsum, clays, dolostones and limestones, Jurassic dolomites, Cretaceous clay and limestone, Oligocene siliciclastic flysch (Numidian flysch), continental Neogene siliciclastic sediments and Quaternary and recent fluvial deposits in the plain.

The study basin contains several Pb–Zn ore deposits, Hg mine, including Oued Maaden, Thuburnic and Arko El Hafer (Fig. 1; Decrée et al., 2013; Jemmali et al., 2013; Sainfeld, 1952; Slim-Shimi & Tlig, 1993). According to Rouvier et al. (1985), two distinct types of Pb–Zn deposits can be found. The first Pb–Zn mineralisation includes the enrichment of As and Sb and occurs in the continental Neogene sequences. The second one shows the enrichment of As and Hg and is found in fractures locally injected with Neogene volcanic rocks. The Oued Maaden mine constitutes the most economic ore deposits of the study basin. Mineralisation is found at Oued Maaden along the NE–SW Groura and Ferza trending faults developed within the Triassic dolostone and the Campanian–Maastrichtian limestone rocks (Jemmali et al., 2013). The main ore minerals are galena, sphalerite, tetrahedrite, malachite, cinnabar and As–Sb-rich sulfosalts alongside other elements, such as Ag and Cd (Jemmali et al., 2013; Sainfeld, 1952; Slim-Shimi & Tlig, 1993). These minerals commonly occur along the main vein and stockwork systems. The Oued Maaden mine was extensively exploited from 1900 to 1955 and produced over 11,500 tons of Pb and 89 tons of Cu concentrates with an Ag concentration of 350 mg kg^−1^ (Sainfeld, 1952). Finally, in the southwest part of the study area is localised the Ghardimaou plain that is one of the most fertile soils in Tunisia, used for intensive agriculture mostly consisting of cereals, vegetables, orchards and fodder crops.

### Sample collection and analytical procedure

A stainless-steel shovel and a tube auger were used to collect 156 stream sediment samples (1–1.5 kg of active sediment) from the Oued Rarai basin (Fig. 2) in March 2016. The samples were subsequently stored in polyethylene bags. For each site, three to five sub-samples were collected at the upper 5-cm sediment in a maximum range of 200 m (Salminen et al., 1998) and then pooled together to obtain a homogeneous composite sample. Geographic coordinates of each sampling site were recorded on a field sheet.

In the laboratory, sediment pH was measured in a 1:5 sediment–water suspension using a METTLER TOLEDO pH Electrode (LE427-IP67).

Bulk sediment samples were air-dried, sieved to 2 mm with a stainless-steel sieve and manually crushed in an agate mortar. Sub-sample (0.5 g) was heated in a mixture of 5 mL of HNO_3_, 10 mL of HClO_4_ and 10 mL of HF to fuming and taken to dryness (Khalil et al., 2013).

Total concentrations of Ag, As, Cd, Co, Cr, Cu, Hg, Ni, Pb, Sb, V and Zn were determined using inductively coupled plasma–atomic emission spectroscopy (Ultima C HORIBA-Jobin Yvon) at the National Office of Mines in Tunis, Tunisia. The quality of the analytical procedure for the total trace element concentrations was checked by analysing the stream sediment reference samples (from National Office of Mines, Tunisia) and eight replicate samples for which the relative standard deviations (%RSDs) were less than 10% for the trace elements. According to the trace element, the recovery $({Concetration}_{measured}/{Concentration}_{certified}\times 100)$ was in the range of 84–112% (supplementary file S1).

### Geochemical background and environmental indices

#### Geochemical background

The geochemical background is considered to be an element or compound concentration unaffected by anthropogenic inputs (Baize & Sterckeman, 2001). The threshold value is defined as an upper limit without an anthropogenic influence (Matschullat et al., 2000; Taylor & Kesterton 2002; Reimann & Garrett, 2005).

In this study, the geochemical background thresholds were determined using a multi-element geochemical data set of Ghardimaou topographic map of scale 1:50.000 collected during a regional geochemical survey by the Tunisian National Office of Mines (Loukil, 1994). For the survey a total of 1090 samples with a sample density of 1–2 samples per km^2^ were analysed for trace elements using the same analytical procedures previously discussed. The average per cent recovery was between 86 and 108% for all the investigated trace elements. The distribution function method was used to estimate the geochemical background distributions due to its robustness (Matschullat et al., 2000). This statistical procedure is based on the assumption that the first part of the data set (i.e. from the minimal value to the median value) is not influenced by anthropogenic inputs. In this study, the second half of the distribution was built by ‘mirroring’ each value against the median by adding the distance from the value to the median. The mean and standard deviation (*σ*) scores of the newly obtained data set were used to calculate the geochemical background threshold: mean + 2*σ* (Matschullat et al., 2000). In the proposed case study, not only the data set from minimal to median value, but also the data set from median to maximum value would be considered as data set that is also not influenced by anthropogenic activities.

#### Geoaccumulation index

The geoaccumulation index (Igeo) was used to evaluate the level of trace element contamination in sediments. The Igeo was originally defined by Müller (1979) and widely used in environmental studies (Barbieri et al., 2014, 2018; Wang et al., 2014). This index was calculated for each trace element as follows:

$${\mathrm{Igeo}=\mathrm{log}}_{2}\left(\frac{{C}_{x}}{1.5{B}_{x}}\right)$$

where ${C}_{x}$ is the measured metal concentration; ${B}_{x}$ is the geochemical background value of the element of concern using the upper continental crust (UCC; Taylor & McLennan, 1985); and the constant 1.5 is the geochemical background matrix correction factor used to minimise possible variations in the background values due to natural sources. Igeo was classified into seven classes of contamination level (supplementary file S2), as described by Müller (1979).

#### Potential ecological risk index

To assess the ecological risk of trace elements in analysed sediments, the potential ecological risk index (RI), originally introduced by Hakanson (1980), was calculated using the sum of risk factors of individual trace elements. This index was calculated as follows:

$$\text{RI = }\sum_{{\text{i}}= \text{1} }^{\text{n}}{{\text{E}}}_{\text{i}}$$

where ${\text{E}}_{\text{i}}$ is the potential ecological risk factor for each trace element *i* defined as:

$${\text{E}}_{\text{i}}\text{ } = {\text{T}}_{\text{i}}\frac{{\text{C}}_{\text{i}}}{{\text{B}}_{\text{i}}}$$

where ${\text{T}}_{\text{i}}$ is the toxic–response factor for each trace element I, as calculated by Hakanson (1980) (i.e. Hg = 40, Cd = 30, As = 10, Pb = Cu = Ni = Co = 5, Cr = V = 2, and Zn = 1; ${\text{C}}_{\text{i}}$ is the measured concentration of the trace element i; ${\text{B}}_{\text{i}}$ is the background value of the trace element i using UCC data set (Taylor & McLennan, 1985; Wedepohl, 1995). Hakanson (1980) defined four levels of RI and five levels of E_*i*_(supplementary file S2).

### Source identification and apportion contributions

Principal component analysis (PCA), a common traditional multivariate statistical tool, was used to classify different groups of trace elements in stream sediments regarding their geochemical patterns (Chen et al., 2016; Varol, 2011). Recently, many approaches have been proposed to identify contamination sources and apportion contributions of each identified source (Ribeiro et al., 2010; Wang et al., 2014).

### Data analysis

All descriptive statistics were performed using SPSS 20 (IBM Corp., Armonk, NY). Multivariate analyses were performed with R 3.5.1 (R Foundation for Statistical Computing, Vienna). *FactoMineR* and *factoextra* packages were used to perform the PCA.

## Results and discussion

### Stream sediment pH

The solubility of trace elements highly depends on the pH values of sediment: it generally tends to increase at lower pH and decrease at higher pH (Rieuwerts et al., 2015; Wang et al., 2015). In this study, the pH values of sediment samples ranged from 7.4 to 8.5, with a mean value of 8.1, which implies moderately alkaline conditions for sediment samples. These results are in agreement with data from northern Tunisia (Boussen et al., 2013; Pascaud et al., 2015).

Based on the *p values* and Pearson correlation (Table 1), sediment pH did not correlate with the trace element concentrations. Due to the moderately alkaline environment, pH has a limited impact on the distributions of trace elements in stream sediments, substantially limiting their mobility (Boussen et al., 2013; Pascaud et al., 2015).

**Table 1:** Pearson correlation coefficients **(1)** and p values **(2)** between trace element concentration and pH in stream sediment samples from the Oued Rarai basin

pH	Ag	As	Cd	Cr	Cu	Hg	Ni	Pb	Sb	V	Zn
(1)	0.14	0.03	0.14	0.12	0.13	0.12	0.06	0.15	0.15	0.12	0.09
(2)	0.088	0.72	0.088	0.14	0.11	0.14	0.47	0.067	0.067	0.14	0.27

### Trace element concentrations and contamination levels

#### Antimony

Antimony concentrations ranged from 0.02 to 297 mg kg^−1^ (Table 2). In the study basin, 25% of samples were higher than the upper limit of the natural background concentrations (34.8 mg kg^−1^), and 62% of samples exceeded the Dutch standard (15 mg kg^−1^). The Sb median of 19.2 mg kg^−1^ was higher than the UCC value. The distribution of Sb in the stream sediment samples was similar to what was observed for As. Most of the study basin was highly contaminated (Igeo > 5) by Sb, supporting a large Sb enrichment in the whole basin. The median concentration of Sb in stream sediments in the study basin was 19 mg kg^−1^, higher than that found in the contaminated mining sites (range from 5 to 9.8 mg kg^−1^, respectively, reported by Darwish, 2016; Cortada et al., 2018).

**Table 2:** Summary statistics of trace element concentrations in stream sediment samples from the Oued Rarai basin plus comparisons with indicative values and geochemical background thresholds

Element	Summary statistics (mg kg^−1^, except for CV* in %)			Indicative values (mg kg^−1^)					Geochemical background threshold (mg kg^−1^)
	Mean	SD*	CV*	Median	Min	Max	UCC^a^	NIL^b^	
Ag	0.36	1.12	309	0.05	0.05	9.40	0.05	15	0.30
As	85.8	170	198	45.3	0.50	1490	1.5	55	47.0
Cd	0.64	1.14	177	0.05	0.05	8.01	0.098	12	1.01
Cr	57.4	31.4	54	55.5	3.46	198	35	380	40.1
Cu	22.6	10.9	48	21.0	3.85	66.8	25	190	23.2
Hg	1.54	5.56	361	0.05	0.05	54.4	0.056	10	0.50
Ni	36.7	14.2	38	36.1	5.28	87.6	20	210	43.1
Pb	147	531	360	30.4	2.90	5150	20	530	36.3
Sb	32.1	45.2	140	19.0	0.02	297	0.2	15	34.8
V	57.0	27.8	48	52.8	6.12	148	60	250	49.0
Zn	180	255	141	124	23.2	2610	71	720	144

*SD standard deviation; *CV* coefficient of variation (%); *nd* not determined

^a^upper continental crust (Taylor & McLennan, 1985, except for Hg: Wedepohl, 1995)

^b^Dutch standard for toxic elements in stream sediments (ESDAT, 2013)

#### Arsenic

The concentration of Arsenic varied widely from 0.5 to 1490 mg kg^−1^; (Table 2), with 46% of samples having a higher concentration than the upper limit of the natural background. Overall, more than 62% of all sampling sites exceeded the Dutch standard (55 mg kg^−1^). The As median was 45.3 mg kg^−1^, largely greater than those found in the contaminated mining sites (Cortada et al., 2018; Darwish, 2016), indicating that a large part of the study basin was enriched in As (Igeo > 5). Arsenic could be released from sulphide minerals (e.g. galena, sphalerite, tetrahedrite, malachite, cinnabar and As–Sb-rich sulfosalts) during mining operation and weathering.

Accordingly, the highest values of As were in correspondence with the mining activities and the siliciclastic sediments of the continental Neogene that covering hilly areas surrounding the Oued Rarai plain.

#### Cadmium

Cadmium concentrations in the studied sediments ranged between 0.05 and 8.01 mg kg^−1^ (Table 2), with 70% of stream sediment samples characterised by values below 0.1 mg kg^−1^, which represents the upper limit of the natural background in the study basin. Cadmium concentrations in all samples were within the limits given by the Dutch standard (12 mg kg^−1^). As observed for Ag, the highest values of Cd were measured in stream sediments from Oued Rarai, and its tributaries were the drained mineralised area of the Oued Maaden’s abandoned mine, meaning that Cd concentrations were mostly related to mineral occurrences and mining wastes. Cadmium was abundant in ore minerals at Oued Maaden (Jemmali et al., 2013; Slim-Shimi & Tlig, 1993). Across Oued Maaden, Cd concentrations in sediments (range from 4 to 5 mg kg^−1^) did not show a downstream decrease, especially in plain samples compared with numerous existing studies (Pascaud et al., 2015; Wolfenden & Lewin, 1977). The Cd increase could be attributed to the considerable reactivity and mobility of Cd^2+^ and Cd (II) species in the solid phase. High Cd contamination (Igeo > 5) occurred in the drainage basin of the Oued Maaden mining site and Oued Rarai plain. Similar average values were recorded in sediments near contaminated sites (Salvarredy-Aranguren et al., 2008; Yacoub, 2014).

#### Chromium

Chromium concentrations in sediments ranged from 3.46 to 198 mg kg^−1^ (Table 2), with an upper limit of natural background (40.1 mg kg^−1^) close to the corresponding UCC value (35 mg kg^−1^). All samples had Cr concentration under the Dutch standard (380 mg kg^−1^). The highest values occurred mainly in clayey deposits in hilly areas and alluvial plain. Moderate contamination (1 < Igeo < 3) characterised most samples from the study basin. Chromium concentrations observed in the Oued Rarai basin fell within the range found in the literature (range from 39.80 to 148 mg kg^−1^, respectively, reported by Zuluaga et al., 2016 and Shruti et al., 2013).

#### Copper

Copper concentrations in Oued Rarai sediments ranged from 3.58 to 66.8 mg kg^−1^ (Table 2), with an upper limit of natural background of 23.2 mg kg^−1^, relatively comparable to the corresponding UCC value (25 mg kg^−1^). In the study basin, 9% of samples exceeded the upper limit of the natural background, and all stream sediment samples had Cu concentration under the Dutch standard (190 mg kg^−1^). The highest Cu concentrations were found in the drainage basin of the Oued Maaden mining site, mineralised areas (e.g. Arko-Hafer, Thuburnic). Low contamination (Igeo < 1) characterised the whole study basin. Copper median concentrations in this study were significantly lower (i.e. 5–8 times) than concentrations observed in others mining districts (Cortada et al., 2018; Salvarredy-Aranguren et al., 2008).

#### Lead

Lead concentrations in Oued Rarai sediments ranged from 2.9 to 5150 mg kg^−1^ (Table 2), with an upper limit of natural background of 36.3 mg kg^−1^, slightly above the corresponding UCC value (20 mg kg^−1^). In the study area, 41% of samples were above the upper limit of the natural background. Although the mean concentration of Pb was below the Dutch standard (530 mg kg^−1^), Pb concentrations in seven stream sediment samples surpassed the Dutch standard, with an average factor of 3.7. Elevated Pb concentrations were observed in the drainage basin of the Oued Maaden mining site. Lead concentrations showed a similar pattern as observed for Hg (*r*^2^ = 0.85); Pb concentrations in sediments did not show a downstream decrease (Hudson-Edwards et al., 2001; Lecce & Pavlowsky, 2014). Indeed, high Pb contamination (Igeo > 5) was in correspondence with that of Hg: the drainage basin of the Oued Maaden mining site and tributaries cutting NE–SW trending faults. If the Pb enrichment around the mining site was probably due to the ore-related activities as reported by several studies (Oyarzun et al., 2011; Pascaud et al., 2015; Salvarredy-Aranguren et al., 2008), the heterogeneity of Pb concentrations observed in plain samples implied the presence of additional sources along the river. The lead median value was twice higher than that found in the stream sediments from Oued Mellègue, northern Tunisia (14 mg kg^−1^, Mlayah et al., 2009). In contrast, higher concentrations of Pb (700 mg kg^−1^) were reported in the sediments of Oued Ghezini, which drains the Jalta Pb–Zn mine in northern Tunisia (Pascaud et al., 2015).

#### Mercury

Mercury concentrations in the sediments from the Oued Rarai basin ranged from 0.05 to 54.4 mg kg^−1^ (Table 2), with most of the studied samples (80%) exhibiting values below 0.5 mg kg^−1^, which represents the upper limit of the natural background in the study basin. Only 10 stream sediment samples had concentrations of Hg, which exceeded the Dutch standard (10 mg kg^−1^). The highest values of Hg concentrations were observed downstream a large abandoned open-pit developed in Hg deposits. Therefore, the high Hg concentrations could be attributed to the ore-related operations of the former Hg mine, which led to particulate inputs in Oued Maaden. As observed for Cd, Hg concentrations in sediments from Oued Maaden dropped dramatically in the sediment samples located less than 80 m from the mining sites but then increased up to 21 mg kg^−1^ in samples collected along the lower stretch of Oued Maaden before the Ghardimaou plain.

The lack of a decrease in the contaminant concentration downstream the pollution source is typical for historical primary pollution. The cleaner particulates from reaches upstream the historical sources have continuously diluted the primary contamination. It is noteworthy that Hg concentrations were below the detection limit (0.05 mg kg^−1^) along Oued Rarai, except for two samples located at the confluence of Oued Maaden. Tributaries cutting NE–SW trending faults also showed significant Hg concentrations. The drainage basin of the Oued Maaden mining site and tributaries cutting some NE–SW trending faults were characterised by high Hg contaminations (Igeo > 5). The mercury median value was 10–50 times lower than those observed near the Punitaqui Cu-Au-Hg mine in Chile (Hg concentration in stream sediment range from 0.2 to 3.6 mg kg^−1^,Higueras et al., 2004) and Kalecik and Palawan Quicksilver Hg mines in the Philippines (range from 3.5 to 52 mg kg^−1^, Gray et al., 2003).

#### Nickel

Nickel concentrations in samples ranged from 5.28 to 87.6 mg kg^−1^ (Table 2), below the Dutch standard (210 mg kg^−1^). Approximatively, 28% of samples presented values higher than the upper limit of the natural background (43.1 mg kg^−1^). The Ni median value was (36 mg kg^−1^), higher than the corresponding UCC value (20 mg kg^−1^), indicating a substantial Ni enrichment. The whole Oued Rarai basin was mainly characterised by a moderate Ni contamination, with the highest contamination level recorded in correspondence with the Neogene siliciclastic sediments in hilly areas. Nickel concentrations were higher than the values found in the literature (range from 3.5 to 25 mg kg^−1^, respectively, reported by Gray et al., 2003, Morillo et al., 2002 and Cortada et al., 2018). As for the chromium and vanadium concentrations, even in this case, the felsic plugs and mafic dikes, sills and basaltic flows which crosscut the nappe pile of the Kroumirie chain may represent relevant geogenic sources.

#### Silver

In the Oued Rarai basin, Ag concentrations in stream sediments ranged from 0.05 to 9.4 mg kg^−1^ (Table 2), with most of the samples (76.3% of total samples) characterised by concentrations lower than the upper limit of the natural background of 0.3 mg kg^−1^. In total, 23% of stream sediments had concentrations higher than the UCC value (0.05 mg kg^−1^) reported by Taylor and McLennan (1985), and all samples showed Ag concentrations below the intervention value (15 mg kg^−1^) established by the Dutch standard (ESDAT, 2013). The highest concentrations (from 4 to 9.4 mg kg^−1^) were measured in the mineralised area of the Oued Maaden Pb–Zn and Hg mines, where values were up to 184 times higher than those of the UCC. Relatively high concentrations were also observed in the Oued Maaden plain samples. Spatial distribution of Igeo indicated that a large part of the study basin was characterised by high Ag contaminations (Igeo > 5). The high Ag contamination, mainly in correspondence with the drainage basin of the Oued Maaden mining site and Oued Rarai plain. Following numerous previous studies, high Ag concentrations can be related to former mining activities at Oued Maaden, which was responsible for the release of several millions of tons of tailings in the drainage basin (Ferreira da Silva et al., 2015; Oyarzun et al., 2011). Silver concentrations (range from 0.05 to 9.40 mg kg^−1^), however, were 10–100 times lower than those observed in mining sites worldwide (Cortada et al., 2018; Ferreira da Silva et al., 2015; Oyarzun et al., 2011).

#### Vanadium

The concentration of vanadium varied widely from 6.12 to 148 mg kg^−1^, with an upper limit of the natural background of 49 mg kg^−1^ (Table 2), slightly lower than the corresponding UCC value (60 mg kg^−1^). In total, 53% of samples exceeded the upper limit of the natural background, and all samples had a V concentration below the Dutch standard (250 mg kg^−1^). The highest values of V concentrations were observed close to the Oued Maaden mine. A low V contamination (Igeo < 1) characterised the whole study basin. Median concentrations of V were relatively comparable to those found in the literature (range from 45 to 53 mg kg^−1^, respectively, reported by Zuluaga et al., 2016; Shruti et al., 2013).

#### Zinc

Zinc concentrations ranged from 23.2 to 2610 mg kg^−1^, with an upper limit of the natural background of 144 mg kg^−1^, higher than the UCC value (71 mg kg^−1^) (Table 2). In this study basin, 35% of sediments exceeded the upper limit of the natural background, and only four samples were higher than the Dutch standard (720 mg kg^−1^). The highest Zn concentrations were mainly found in the stream sediment samples from the Oued Maaden’s abandoned mine. Some tributaries cutting the NE–SW trending faults also revealed significant Zn concentrations. Distribution of the Igeo displayed high Zn contamination (Igeo > 3) occurring close to ore deposits, showings and tectonic contacts, while the rest of the study basin was weakly to moderately contaminated (Igeo < 2). The measure Zn concentrations were similar to the average values observed in mining sites in Peru (160 mg kg^−1^, Yacoub et al., 2014), Chile (180 mg kg^−1^ Higueras et al., 2004) and Spain (155 mg kg^−1^Arias et al., 2012).

### Risk assessment

**Fig. 3:**
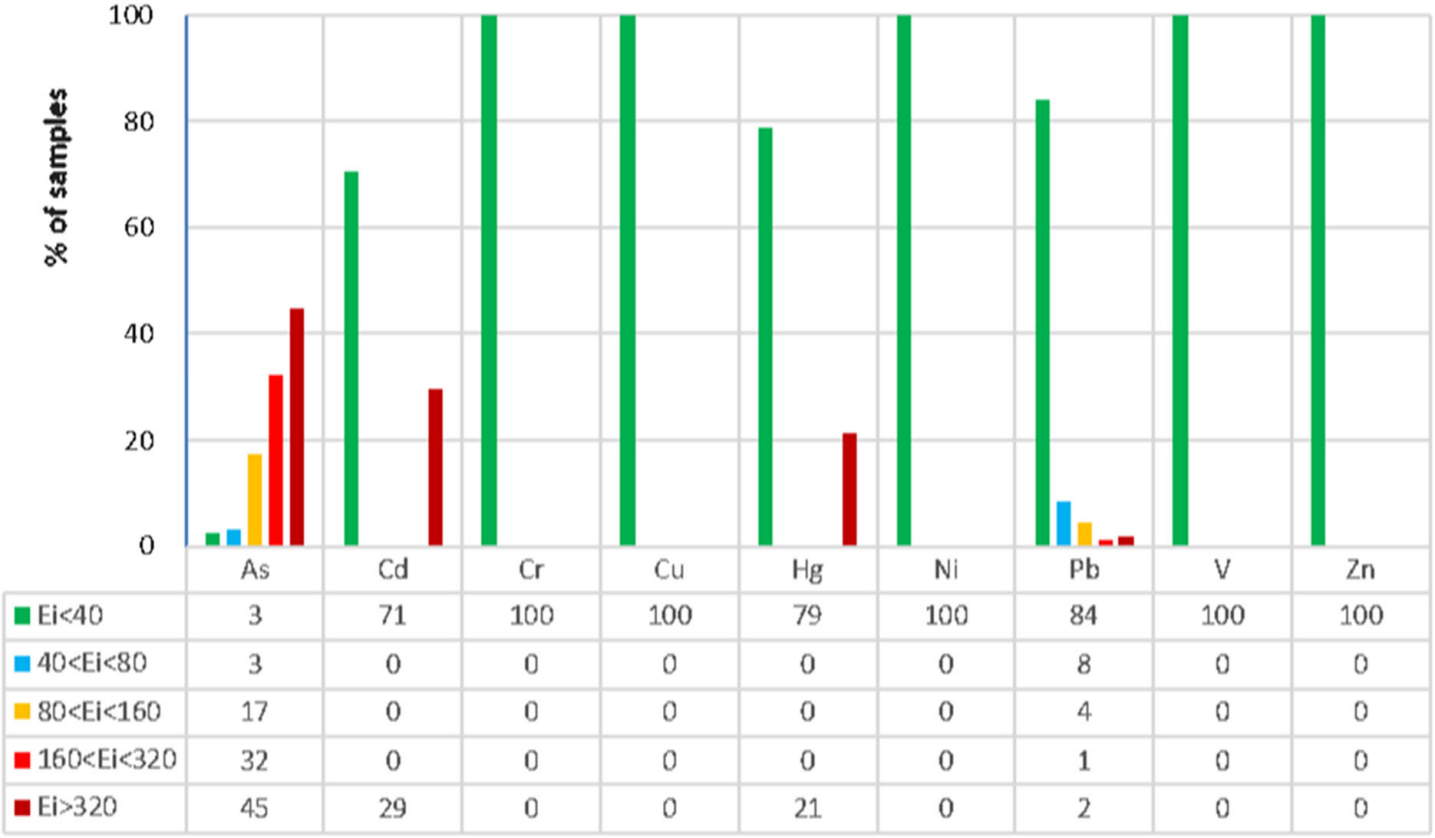
Potential ecological risk factor (*Ei*) of nine trace elements in the study basin

The potential ecological risk was assessed using the RI to identify trace elements posing environmental risk (Fig. 3). On average, *E*_*i*_ values were maximum for As with a median of 302, indicating a very high to serious ecological risk (> 160). Such high values were found in most sampling sites (77% of the data set). Arsenic contamination was more severe in the Oued Rarai basin than other regions of Tunisia where the median *E*_*i*_ of As reached three times that in the Nefza region (Ayari et al., 2016), which corroborates the Igeo results. Considering the absence of Sb and Ag in the current *E*_*i*_ calculations, As appeared as the principal trace element responsible for the ecological risk in the study area, followed by Cd, Hg and Pb. Indeed, E_i_ factor reached locally very high values for Cd, Hg and Pb, with 29%, 21% and 16% of data points being > 40, respectively (Fig. 3). Conversely, E_i_ for Cr, Cu, Ni, V and Zn never exceeded 40.

Considering all studied trace elements, RI results showed that 81% of all sampling sites posed considerable to very high ecological risks (RI > 300), which were more critical than other regions in northern Tunisia (Ayari et al., 2016). We assume that the Oued Rarai basin has had significant toxicological and ecotoxicological impacts on natural ecosystems. Sediment samples with higher ecological risks were found in the tributaries crossing the tectonic contact and the drainage basin of the Oued Maaden mine. These observations agreed with the distribution patterns of concentrations and Igeo of trace elements. Thus, both natural lithogenic and mining activities were considered the main factors controlling trace element contamination.

### Source identification and apportion contributions

**Fig. 4:**
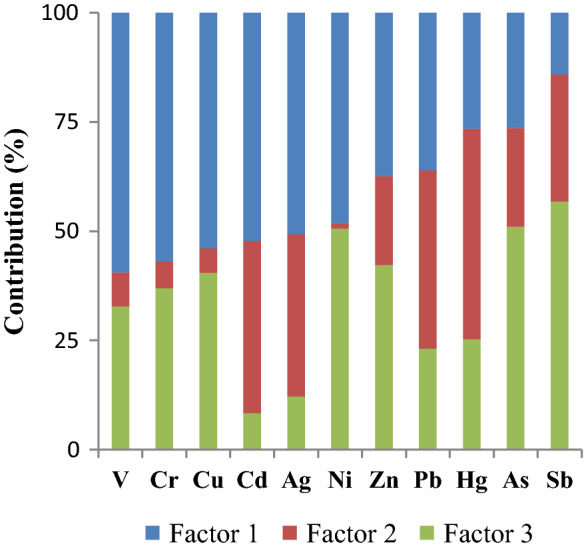
The contribution of the three identified factors

A PCA was applied to investigate the correlations among trace element concentrations in sediments from the Oued Rarai basin in reduced dimensions. The resulting first three principal components accounted for 74% of the total data variance (Table 3). The first principal component (PC1, 34% of the total data variance) explained Cr, Ni, V and Cu with the strongest positive scores and Hg with the negative ones (Table 3 and Fig. 4). In this case, the felsic plugs and mafic dikes, sills and basaltic flows which crosscut the nappe pile of the Kroumirie chain may represent relevant geogenic sources.

**Table 3:** Scores of the first three principal components (PC) from the PCA based on trace element concentrations in stream sediments from the Oued Rarai basin

Element	PC1	PC2	PC3
V	0.91	0.20	− 0.07
Cr	0.89	0.05	− 0.11
Ni	0.86	− 0.42	− 0.05
Cu	0.85	− 0.07	− 0.07
Hg	− 0.63	0.22	− 0.29
Cd	0.05	0.91	− 0.09
Ag	0.07	0.78	− 0.19
As	− 0.04	− 0.58	− 0.28
Sb	− 0.42	− 0.82	0.05
Pb	− 0.18	0.38	0.83
Zn	0.31	− 0.20	0.82
Eigenvalue	3.79	2.90	1.59
Data variance (%)	34	26	14

**Fig. 5:**
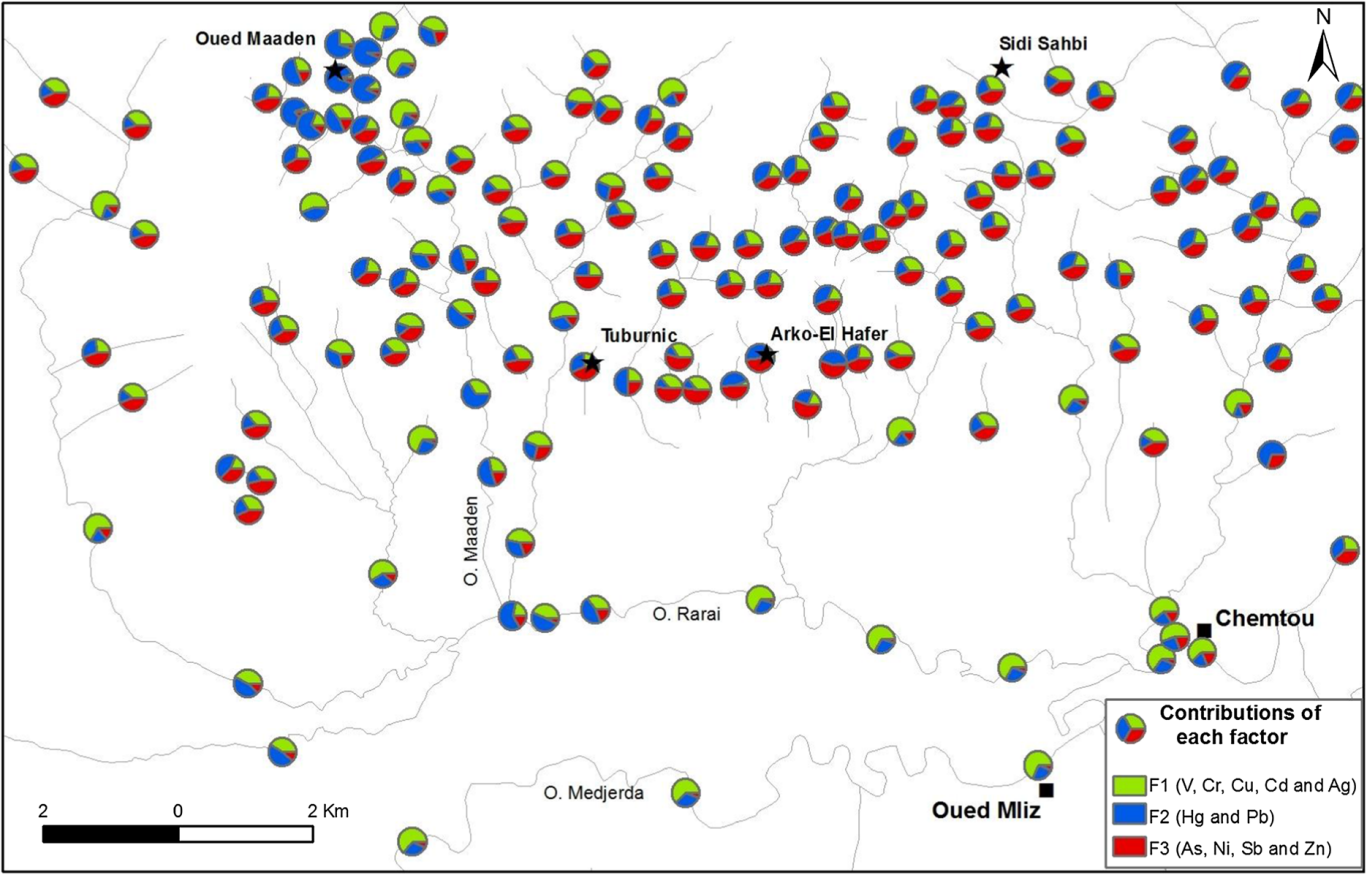
The contribution of the three identified factors to the investigated trace elements in the Oued Rarai basin

Negative loading of Hg in PC1 reflected different sources and depositional environments of Hg and Cr–V–Cu–Ni. Historically, the Oued Maaden area included an ancient Hg mine, suggesting that Hg in sediments can be attributed to mining operations and the weathering of abandoned wastes (Higueras et al., 2004; Gemici et al., 2013). The second principal component (PC2, 26% of the total data variance) explained Ag and Cd with positive scores and As and Sb with negative ones (Table 3 and Fig. 4). This last grouping, as well as high concentrations previously reported for As and Sb, agreed with data from the National Office of Mines, Tunisia, and other studies (Rouvier et al., 1985), suggesting that northwest of Tunisia is characterised by As and Sb geochemical haloes (Fig. 5). Indeed, northern Tunisia contains several Pb–Zn deposits with As and Sb enrichments associated with Neogene siliciclastic sediments, such as Bou Aouane, Bazina and La Semene (Rouvier et al., 1985). According to Bouaziz et al. (2002), the paleocatchment of Oued Rarai was coincident in the depositional environments of detrital sediments during Miocene. In the sediments, As and Sb were better adsorbed by oxyhydroxides than by clay minerals, as reported in many studies (e.g. Leuz et al., 2006; Smedley & Kinniburgh, 2002).

The inverse correlation between As–Sb and Ag–Cd supports the presence of distinct sources and patterns. First, Ag and Cd could be controlled by historical mining activities occurring in the study basin, because they are found as minor components in ore minerals (Slim-Shimi & Tlig, 1993). Second, Ag and Cd were more associated with fine-grained particles deposited in the alluvial plain, as highlighted by many authors (e.g. Cossa et al., 2014; Zuzolo et al., 2016).

Finally, the third principal component (PC3, 14% of the total data variance) explained both Pb and Zn with positive scores (Table 3 and Fig. 4). The highest concentrations for both Pb and Zn occurred in the drainage basin of the Oued Maaden mine and along some tributaries cutting the tectonic contact in the hilly area. Therefore, Pb and Zn (Fig. 5) seemed to be mainly controlled by mining operations, especially downstream mining sites (Arias et al., 2012; Salvarredy-Aranguren et al., 2008).

Past flooding events had served to deposit coarse fractions containing Hg and Pb in the alluvial plain. The contaminated sediments in the lower land were masked by fine particles rich with V, Cr, Cu, Cd and Ag. The contaminated sediments were deposited during the low-energy hydrological environment while, in the relatively higher land, the coarse sediments remained on the surface. Today, deeper contaminated sediments can be resuspended by the surface run-off during heavy rainfalls, constituting a potential secondary source for trace element contamination in the floodplain (Axtmann & Luoma, 1991; Moore et al., 1989). Sediments deposited in the higher land were eroded during severe flood discharges and were returned to the river sediments by tributaries (Macklin & Lewin, 1989; Miller et al., 1999). These findings confirm that the erosion and deposition of materials, deriving from rocks hosting mineralised vein bodies exposed in the paleo-watershed system, were the dominant sources for the higher concentrations of these elements.

## Conclusions

A total of 156 stream sediment samples collected from the Oued Rarai basin in northern Tunisia were analysed to determine the total concentration of trace elements (As, Cd, Cr, Cu, Ni, Pb, Sb, V and Zn) to identify both contamination levels and sources of these potentially toxic elements. Based on total concentrations and Igeo, the stream sediments were contaminated by trace elements to different degrees. The contamination level reported in this study indicates a non-negligible potential ecological risk, mainly related to sediment transport along the river. Antimony and arsenic have been identified as principal contaminants exceeding the Dutch standard for 62% and 40% of all sampling sites, respectively. Results from correlation analysis and principal component analysis revealed three main geochemical associations related to lithologic, tectonic and anthropogenic sources. V, Cr, Ni and Cu mainly originated from natural bedrock and soil. Ag and Cd were more controlled by mining enrichments. Ag, Hg and Pb were mostly influenced by the ancient ore-related activities at the Oued Maaden site and NE–SW trending faults. Finally, Sb, As, and Zn were largely controlled by the siliciclastic continental Neogene sequences.

The concentrations and patterns of trace elements in the study basin were mostly controlled by the physical and chemical dynamics of the watershed system, lithological properties, mineralisation, tectonic settings and mobilisation of subsurface sediments. The large flooding events similar to those in the Mediterranean region in recent years play a crucial role in the remobilisation of some trace elements from historically mining polluted deposits, whereas present-day pollutants are primarily transported during moderate and low flows. To prevent river pollution and improve the watershed ecosystem health, it is important to understand clearly the contamination characteristics of trace elements in sediments, soils and vegetation and target their potential source.

The current findings could inform environmental risk management of the studied contaminants on a catchment scale.

## Supplementary Information

Below is the link to the electronic supplementary material.

Supplementary file1 (DOCX 13 KB)

Supplementary file2 (DOCX 14 KB)
