# Supplementary material for: Trace element contamination in the mine-affected stream sediments of Oued Rarai in north-western Tunisia: a river basin scale assessment
Source: Environ Geochem Health. 2021 Mar 26;43(10):4027–42. doi: 10.1007/s10653-021-00887-1 (PMC8473341; doi:10.1007/s10653-021-00887-1)
Supplement: Supplementary file 1 — Supplementary file1 (DOCX 13 KB) [file 10653_2021_887_MOESM1_ESM.docx]

*S1. Analytical quality of the applied method*

| Element | Detection limit (mg kg^−1^) | Recovery (%) |
| --- | --- | --- |
| Ag | 0.05 | 86 |
| As | 0.5 | 92 |
| Cd | 0.05 | 105 |
| Cr | 0.5 | 103 |
| Cu | 0.1 | 95 |
| Hg | 0.05 | 84 |
| Ni | 0.5 | 92 |
| Pb | 0.5 | 112 |
| Sb | 0.02 | 88 |
| V | 0.5 | 108 |
| Zn | 0.5 | 105 |
