# Supplementary material for: Trace element contamination in the mine-affected stream sediments of Oued Rarai in north-western Tunisia: a river basin scale assessment
Source: Environ Geochem Health. 2021 Mar 26;43(10):4027–42. doi: 10.1007/s10653-021-00887-1 (PMC8473341; doi:10.1007/s10653-021-00887-1)
Supplement: Supplementary file 2 — Supplementary file2 (DOCX 14 KB) [file 10653_2021_887_MOESM2_ESM.docx]

*S2. Degree of trace element contamination according to Igeo (Müller, 1979), potential ecological RI (Hakanson, 1980) and potential ecological risk factor (Ei, Hakanson, 1980)*

| **Index** | **Value** | **Degree of contamination** |
| --- | --- | --- |
| **Igeo** | <0 | uncontaminated |
|  | 0–1 | uncontaminated to moderately contaminated |
|  | 1–2 | moderately contaminated |
|  | 2–3 | moderately to strongly contaminated |
|  | 3–4 | strongly contaminated |
|  | 4–5 | strongly to extremely contaminated |
|  | >5 | extremely contaminated |
| **RI** | <150 | low ecological risk |
|  | 150–300 | moderate ecological risk |
|  | 300–600 | considerable ecological risk |
|  | >600 | very high ecological risk |
| **Ei** | <40 | low ecological risk |
|  | 40–80 | moderate ecological risk |
|  | 80–160 | considerable ecological risk |
|  | 160–320 | very high ecological risk |
|  | >320 | dangerous |
